# Habitat Use and Spatial Variability of Hawkfishes with a Focus on Colour Polymorphism in *Paracirrhites forsteri*

**DOI:** 10.1371/journal.pone.0169079

**Published:** 2017-01-26

**Authors:** Darren J. Coker, Veronica Chaidez, Michael L. Berumen

**Affiliations:** Red Sea Research Center, Division of Biological and Environmental Science and Engineering, King Abdullah University of Science and Technology, Thuwal, Saudi Arabia; Department of Agriculture and Water Resources, AUSTRALIA

## Abstract

Identifying relationships between fishes and their environment is an integral part of understanding coral reef ecosystems. However, this information is lacking for many species, particularly in understudied and remote regions. With coral reefs continuing to face environmental pressures, insight into abundance and distribution patterns along with resource use of fish communities will aid in advancing our ecological understanding and management processes. Based on ecological surveys of hawkfish assemblages (Family: Cirrhitidae) in the Red Sea, we reveal distinct patterns in the distribution and abundance across the continental shelf, wave exposure, and with depth, particularly in the four colour morphs of *Paracirrhites forsteri*. Distinct patterns were observed among hawkfishes, with higher abundance of all species recorded on reefs farther from shore and on wave exposed reef zones. *Cirrhitus spilotoceps* was only recorded on the exposed crest, but unlike the other species, did not associate with live coral colonies. Overall, the most abundant species was *P*. *forsteri*. This species exploited a variety of habitats but showed an affinity for complex habitats provided by live and dead coral colonies. No difference in habitat use was observed among the four colour morphs, but distinct patterns were apparent in distribution and abundance with depth. This study suggests that in addition to *P*. *forsteri* exhibiting diverse colour morphologies, these various morphotypes appear to have corresponding ecological differences in the Red Sea. To better understand this, further studies are needed to identify what these differences extend to and the mechanisms involved.

## Introduction

Understanding the ecological relationships between organisms and their environment forms the basic underpinnings for understanding ecosystems and how these may be altered in the face of disturbance [1,2,3]. Reef assemblages are highly influenced by environmental variables as this determines habitat and food availability, physiological performance, distribution, and abundance. Characterising fish communities and their requirements aids in understanding functional diversity, ecological niches, and assists in effective management of coral reefs [4,5,6].

Environmental variables often exist on a spatial gradient in coral reefs and can influence patterns of assemblage composition for associated organisms along this gradient. For example, turbidity, temperature, sediment load, light levels, salinity, wave energy, nutrients, and benthic composition can impact species distribution and abundance patterns. Studies have shown shifts in various fish taxa with cross shelf gradients, wave energy, and depth [7,8,9,10,11]. This means that environmental heterogeneity produces distinct environmental habitats along a gradient that positively or negatively influence organisms depending on their requirements or adaptations.

The manner in which habitats are partitioned among organisms has direct effects on population densities, species interactions, and the assemblage of ecological communities [12,13,14,15,16]. The various habitat selection strategies employed by organisms may also give us insight into their evolutionary trajectories [14]. Furthermore, the degree of specialisation for any organism lies on a continuum, with generalists using a variety of habitats or resources, specialists using a narrower range of resources, and highly specialised organisms that optimise the use of one or two resources [17]. Understanding this is fundamental to understanding the ecology of coral reefs and how changes in habitat quality will impact associated assemblages.

Reef assemblages are highly influenced by benthic composition and physical structure of the reef as this determines habitat availability, quality and quantity of food, and predator-prey interactions [18,19,20,21,22,23]. Some reef fishes depend directly on live coral for food, but many species also take advantage of the physical and biological structure produced by live and dead structurally complex coral colonies for habitat [24,25,26,27]. Consequently, coral communities can influence the distribution and abundance of fishes, particularly for species that have specialised associations (e.g., [28,29,30,31,32]). Understanding habitat use is important given the increased degradation of coral reef ecosystems throughout the world [33,34,35,36].

Hawkfish (Family: Cirrhitidae) are small mesopredators found throughout tropical reefs [37]. These fishes are commonly observed perched on coral colonies, prey on small fish and invertebrates [37,38,39], and potentially play an important part in coral reef food webs through the transfer of energy to larger piscivores. Although this family is globally widespread, studies on hawkfish are limited, particular in the Red Sea. The Red Sea is one of the warmest and most saline seas on the globe [40,41], and although it boasts high biodiversity and endemism it remains one of the most understudied reef systems [42]. Therefore, this study aimed to describe distributions, abundance patterns, and habitat use of hawkfishes along environmental gradients in the Red Sea. Surveys were conducted across: i) the continental shelf, ii) depth gradients, and iii) exposure gradients to wave energy. The most abundant hawkfish in the Red Sea, *Paracirrhites forsteri*, has been observed to show colour polymorphism throughout the tropics (see [37,43]). Interestingly, observations in the Red Sea have revealed that there are four distinct colour morphs. Therefore, we additionally investigated ecological differences among the colour morphs within this species.

## Methods

### Ethics statement

All fieldwork and data collection was observational and non-extractive. The field studies did not involve endangered or protected species. The research was undertaken in accordance with the policies and procedures of the King Abdullah University of Science and Technology (KAUST). Permissions relevant for KAUST to undertake the research have been obtained from the applicable governmental agencies in the Kingdom of Saudi Arabia.

### Survey sites

**Fig 1:**
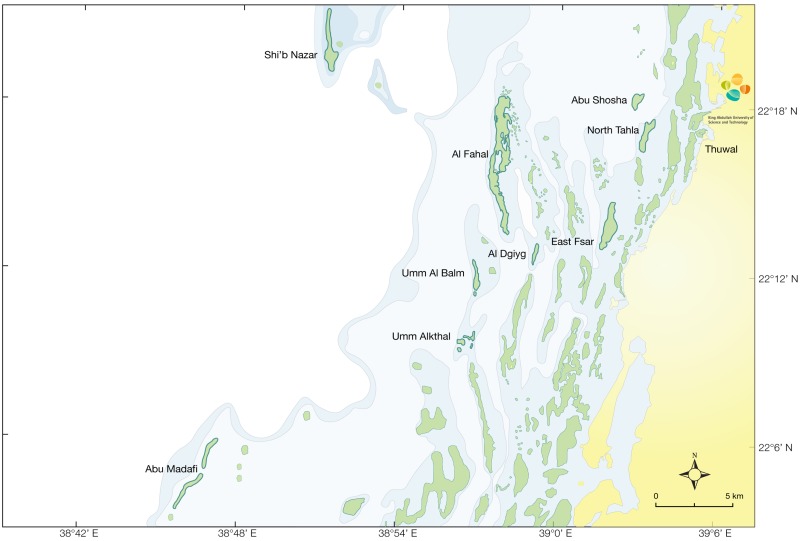
Map of the survey region. The nine surveyed reefs at three different shelf positions (offshore, midshelf, inshore) in the central Red Sea on the Saudi Arabian coast. Bold outlined reefs represent surveyed reefs.

This study was conducted in the central Red Sea off the coast of Thuwal, Saudi Arabia (22.2833° N, 39.1000° E). Nine reefs were surveyed encompassing three inshore (Abu Shosha, East Fsar, North Tahla), four midshelf (Al Dgiyg, Al Fahal, Umm Al Balam, Umm Alkthalal-Kiethl), and two offshore reefs (Abu Madafi, Shi’b Nazar) (Fig 1). Surveys were conducted at a total of 18 randomly selected sites on the west (exposed to the prevailing winds and swell) and east (sheltered) side of all reefs. On offshore reefs, surveys were conducted at 1m (reef crest), 8 m, and 17 m depths on both the exposed and sheltered sides. This was consistent on midshelf reefs, except surveys were not conducted at 17 m on the sheltered side. On the inshore reefs, surveys were conducted at 1m (reef crest) and 8 m on the exposed side and only 1m (reef crest) on the sheltered side. A full coverage of depths was not possible at midshelf and inshore reefs due to their shallow profiles (i.e., the reefs do not always extend to these depths). This configuration of reefs, exposure, and depth was chosen in order to look at the distributions of hawkfish across environmental gradients.

### Fish surveys

**Fig 2:**
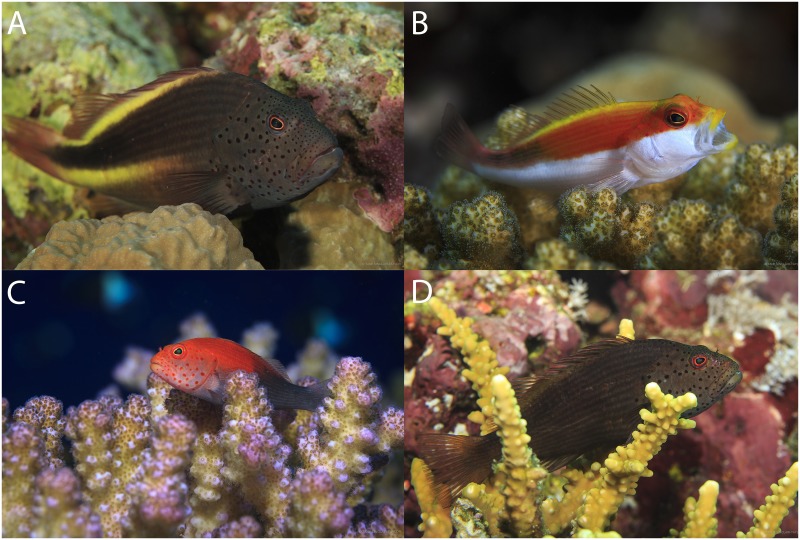
Four colour morphs of the *Paracirrhites forsteri* as observed in the Red Sea. A) Morph, 1 B) Morph 2, C) Morph 3, and D) Morph 4.

Abundance surveys of all hawkfish species were conducted along a 30 m x 4 m belt-transect laid parallel to the reef edge. Habitat use, defined as the substrate that an individual was directly perching/sheltering on when first observed, and estimated total length of each individual fish (TL mm) was recorded for all hawkfishes encountered within a transect. We identified four distinct morphotypes for *P*. *forsteri* based on colour patterns. At present, all colour morphs are considered the same species. Hereafter, we have labeled the colour morphs 1 through 4 (Fig 2). Morph 1 is distinguished by two prominent yellow horizontal stripes across the majority of its body. Morph 2 has a white ventral side and the dorsal half may vary in a combination of yellow, red, and black. Morph 3 exhibits a bright red anterior and dark grey posterior. Morph 4 is completely brown except for a subdued orange tail. In addition to species level abundance and habitat use, all *P*. *forsteri* observed were recorded as one of the four morphs.

### Benthic surveys

To document available habitat for hawkfish and variation across the shelf, exposure, and depth gradient, point intercept transects were conducted along the same transects as the fish surveys. Benthic cover and composition was quantified by identifying the substrate directly underneath each transect every 0.5 m and classified as one of the following fourteen categories: *Acropora*, Pocilloporidae, *Stylophora*, *Millepora*, *Porites*, other live hard coral, *Xeniidae*, other soft coral, dead coral (i.e., dead but structurally intact colonies), rubble, sand, pavement, turf or coralline algae, and other sessile organisms. These benthic categories were chosen because of their relative abundance in the central Red Sea and potential importance for hawkfishes in providing shelter (see [44,45]).

### Microhabitat use

Resource selection ratios were calculated in order to determine if fish were using a benthic category in higher, lower, or equal proportion to availability. This study followed Design I, sampling protocol A from Manly et al. [46]. Ratios were calculated for each benthic category at each shelf position and depth for each species of hawkfish. In addition, habitat selectivity was also calculated for each color morphotype for *P*. *forsteri*. Habitat selection ratios for each reef site were standardised to equal 1 using Manly’s standardised selection ratio (*B*). This value is an index of how likely a category would be utilised if all categories were available at equal capacities. Alpha values were adjusted for pairwise comparisons using Bonferroni-corrected 95% confidence intervals [47]. A confidence interval that encompasses 1 means the microhabitat is being used in proportion to its availability. An interval that is <1 means the microhabitat is underused or avoided and an interval >1 means the microhabitat is chosen or elected for. Data adequate for analysis using Manly’s standardised selection ratios were obtained at 1 m (reef crest) and 8 m (reef slope) depths on the exposed side of reefs but not in sheltered habitats. In addition, all zones that contained only one individual were omitted.

### Activity

While habitat selectivity (see above) was calculated from a first encounter observation and provides an estimate of what habitats are being utilised based on the premise that used habitats may mitigate threats and facilitate daily activities (e.g., feedings, maintaining territory or mates), longer observation periods provide differences in habitat use over time, activity, and spatial movement. Linear regression models were conducted in R (*vegan* package) to identify relationships between distance moved (m) and fish size (TL) for each morph. Observations were conducted on scuba for the four colour morphs of *P*. *forsteri* on the exposed side of Al Fahal reef (midshelf) between a depth of 5 and 15 m. This location and depth range was selected because it contained a high number of all colour morphs along with high coral cover and benthic diversity, and to provide a standardised environment. During a 10 min observation period on randomly selected individuals, the following was recorded: colour morph, total length (TL), habitat association every minute (see above for benthic categories), number of times an individual moved from one perch/shelter to another, and the total distance moved (to the nearest meter). Observations were performed over the course of three consecutive days between the hours of 09:00 and 12:00 in August (end of summer) with each dive conducted at a different site on the reef to eliminate repetitive sampling. Behavioural observations were documented from a distance of approx. 3m and tallied onto a slate. Due to high underwater visibility within this region (often >20m), the distance between the observer and focal fish was adequate to remove observer influences while maintaining an un-obscured view.

## Results

### Abundance patterns

**Fig 3:**
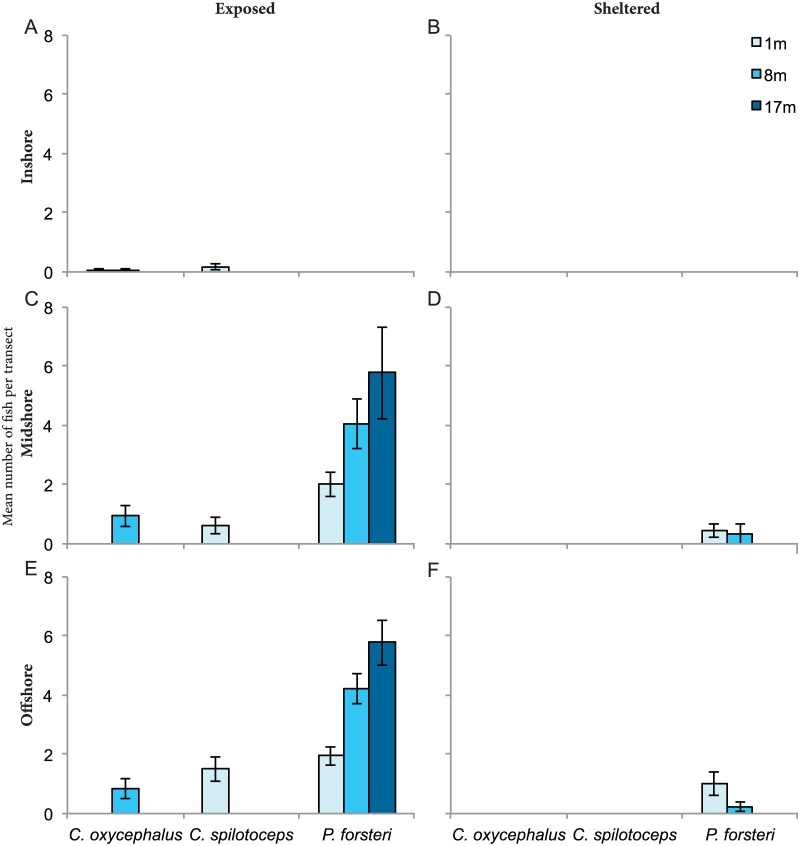
Abundance and distribution patterns of hawkfishes. Distribution densities (mean ± SE) of all observed hawkfish species recorded through underwater visual census across shelf, exposure to wave energy (exposed, sheltered), and depth gradient. A) inshore exposed, B) inshore sheltered, C) midshelf exposed, D) midshelf sheltered, E) offshore exposed, F) offshore sheltered. Surveys were not conducted at 17 m for exposed inshore, sheltered inshore, and sheltered midshelf reefs. It was also not possible to conduct surveys at 8 m on sheltered inshore reefs due to the reefs’ shallow profiles restricting deeper surveys.

Of the four hawkfish species known to exist in the Red Sea, only *Cirrhitus spilotoceps*, *Cirrhitichthys oxycephalus*, and *Paracirrhites forsteri* were recorded in the study area. *Oxycirrhites typus* was not observed on any transects. Overall, *P*. *forsteri* was the most abundant species with a total of 341 individuals recorded compared to 34 and 41 for *C*. *oxycephalus* and *C*. *spilotoceps*, respectively. Although found at all depths and exposure on offshore and midshelf reefs, *P*. *forsteri* was completely absent on inshore reefs. Exposure and depth influenced abundance, with densities up to 6 times higher on the exposed side of reefs and an increase with depth from a mean of 1.9 (± SE 0.2) and 2 (± SE 0.3) individuals per transect at 1 m to a mean of 5.7 (± SE 1.5) and 5.7 (± SE 0.7) at 17 m for the midshelf and offshore reefs, respectively (Fig 3). Abundance was relatively lower for the other two species with less than two individuals per transect at all sites. The endemic Red Sea species *C*. *spilotoceps* was found on reefs at all three shelf positions but only on the exposed crest (1 m). *Cirrhitichthys oxycephalus* displayed a similar pattern except was generally observed at 8 m (Fig 3).

**Fig 4:**
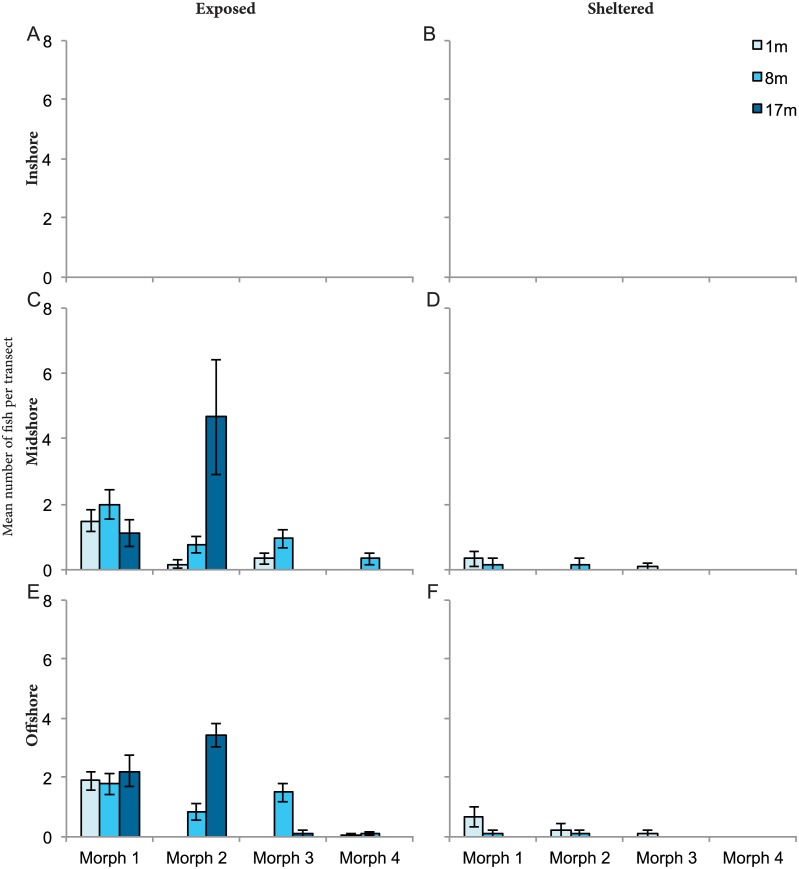
Abundance and distribution patterns of *P*. *forsteri* colour morphs. Distribution densities (mean ± SE) of four *P*. *forsteri* colour morphs recorded through underwater visual census across shelf, exposure to wave energy (exposed, sheltered), and depth gradient. A) inshore exposed, B) inshore sheltered, C) midshelf exposed, D) midshelf sheltered, E) offshore exposed, F) offshore sheltered. Surveys were not conducted at 17 m for exposed inshore, sheltered inshore, and sheltered midshelf reefs. It was also not possible to conduct surveys at 8 m on sheltered inshore reefs due to the reefs’ shallow profiles restricting deeper surveys.

Within the species *P*. *forsteri*, colour morph 1 was the most abundant with a total of 170 individuals (49.9%) followed by morph 2 (109, 31.9%), morph 3 (53, 15.4%), and morph 4 (9, 2.6%) being the least common overall. All four morphs were absent from inshore reefs and more abundant on the exposed side of all reefs than the sheltered side (Fig 4). Among the morphs, morph 1 was present among the broadest range of exposure and depths on the midshelf and offshore reefs, and displayed no trend with depth. Morph 2 was found in the highest densities at a depth of 17 m on the exposed side, peaking at a mean of 4.7 (± SE 1.8) and 3.4 (± SE 0.4) on the midshelf and offshore reefs, respectively (Fig 4), where abundance was 4 to 5 times higher at this depth and exposure than shallower depths. Morph 3 was most commonly recorded at 8 m on the exposed side of the midshelf and offshore reefs (mean 1 ± SE0.3 and 1.5 ± SE 0.3, respectively). Morph 4 was only recorded in low numbers (< 0.1) on the exposed side of the midshelf and outer reefs and never deeper than 8 m.

### Habitat availability

**Fig 5:**
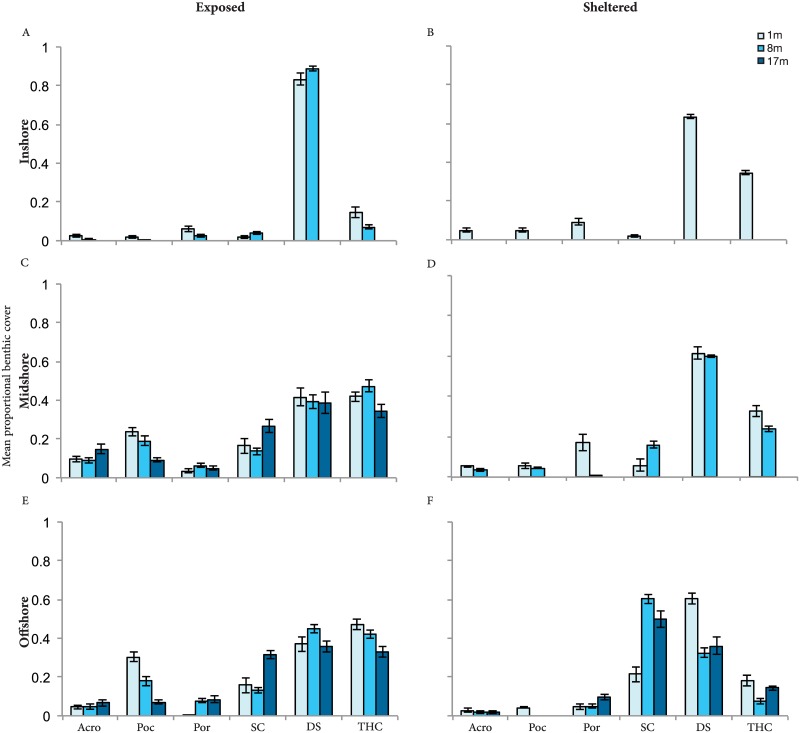
Habitat availability. Proportional benthic cover (mean ± SE) recorded through point intercept transects across shelf, exposure to wave energy (exposed, sheltered), and depth gradient. A) inshore exposed, B) inshore sheltered, C) midshelf exposed, D) midshelf sheltered, E) offshore exposed, F) offshore sheltered. Acro = *Acropora*, Poc = Pocilloporidae, Por = *Porites*, SC = soft corals, DS = dead substrate, THC = total hard corals. Surveys were not conducted at 17 m for exposed inshore, sheltered inshore, and sheltered midshelf reefs. It was also not possible to conduct surveys at 8 m on sheltered inshore reefs due to the reefs’ shallow profiles restricting deeper surveys.

The proportional cover of benthic categories displayed differed across the continental shelf, among exposure within shelf position, and with depth within exposure and position (Fig 5). Exposed sites at midshelf and offshore reefs displayed a higher percentage of total hard coral cover (34–47% and 33–47%, respectively) than exposed sites of inshore reefs (7–14%). At exposed midshelf and offshore reefs, the coverage of Pocilloporidae decreased notably with depth from 1 to 17 m (midshore 23–9%, offshore 30–7%). Similarities were observed between all sheltered sites with high levels of dead substrate (40–60%) and low levels of *Acropora* and Pocilloporidiae (< 5%) (Fig 5). Higher levels of soft coral cover were recorded on offshore reefs with sheltered exposure, with up to 60% of the substrate covered at depths of 8 m and deeper. Exposed inshore reefs had low levels of live coral cover (< 15%) and extremely high levels of dead substrate (approx. 85%). Exposed midshelf and offshore reefs contained up to 50% of total live coral cover dominated by Pocilloporidae and *Acropora*, along with soft corals.

### Microhabitat use

*Cirrhitichthys oxycephalus* was recorded using a range of microhabitats, including live hard coral colonies, soft corals, and dead substrates, but did not display any positive selection for these habitats (Table 1). Interestingly, *Pocillopora* and *Stylophora* were not used, and on the midshelf, *Pocillopora* was underused based on its availability. *Acropora* was unused at offshore sites but used in equal proportion to its availability on midshelf reefs. *Cirrhitus spilotoceps* consistently used only turf algae-covered microhabitats across all shelf positions on the crest and also positively selected for it on offshore reefs (Table 1). A greater number of microhabitats were used at midshelf and offshore reefs than at inshore reefs including live and dead coral habitats.

**Table 1:** Microhabitat selectivity indices for three hawkfish species across an inshore to offshore gradient for 14 categories of substrate.

Species Shelf Position	Depth (m)	n	*Acropora*	*Pocillopora*	*Stylophora*	*Millepora*	*Porites*	Other live hard coral	Xeniidae	Other soft coral	Dead coral	Rubble	Sand	Pavement	Turf algae	Other sessile organisms
***P*. *forsteri***																
Midshelf	Crest	36	= _0.129_	= _0.355_	= _0.132_	U	U	= _0.147_	U	U	= _0.237_	U	U	U	U	U
Midshelf	Slope	73	+_0.232_	= _0.133_	= _0.193_	U	= _0.031_	= _0.053_	U	_- 0.015_	= _0.188_	U	U	U	-_-0.011_	= _0.144_
Offshore	Crest	35	= _0.118_	-_-0.024_	= _0.226_	= _0.123_	= _0.302_	U	U	U	= _0.155_	U	NA	- _0.00_	= _0.053_	NA
Offshore	Slope	76	= _0.121_	+_0.146_	= _0.358_	= _0.062_	_- 0.012_	= _0.069_	U	= _0.023_	= _0.177_	U	NA	U	-_-0.032_	NA
***C*. *oxycephalus***																
Midshelf	Slope	17	= _0.036_	U	U	= _0.349_	U	= _0.202_	U	= _0.03_	= _0.258_	U	U	U	= _0.125_	U
Offshore	Slope	15	U	_- 0.041_	U	= _0.265_	= _0.057_	= _0.132_	U	U	U	= _0.299_	NA	U	= _0.206_	NA
***C*. *spilotoceps***																
Inshore	Crest	3	U	U	U	NA	U	U	U	U	U	U	U	U	= _1.0_	U
Midshelf	Crest	11	U	U	= _0.059_	U	U	U	U	U	= _0.132_	= _0.255_	U	U	= _0.554_	U
Offshore	Crest	27	U	U	U	= _0.274_	U	= _0.101_	U	U	U	U	NA	-_-0.016_	+_0.608_	NA

“=” denotes a category that was used in proportion to its availability,

“+” means the category was used in greater proportion to its availability,

“-” means the category was used at a lower proportion than its availability, and

“U” means the category went unused.

“NA” means the substrate was not present. Subscripts represent Manly’s standardised selection ratio (B), the probability of one resource unit from the given category being selected if all categories were equally available.

Depth of each zone was: crest = 1 m, and slope = 8 m, n = number of individuals analysed.

*Acro* = *Acropora*, *Poc* = *Pocillopora*, *Stylo* = *Stylopora*, *Mille* = *Millepora*, *Por* = *Porites*, OLHC = Other Hard Live Coral, *Xen* = *Xeniidae*, OSC = Other Soft Coral, DC = Dead Coral, Rub = Rubble, Sand = Sand, Pav = Pavement, Turf = Turf Algae, OSO = Other Sessile Organisms.

Generally *P*. *forsteri* used live hard coral and dead coral microhabitats while avoiding soft coral and structurally degraded dead substrate (rubble, pavement, and turf) across all sites (Table 1). Habitat use was consistent among depth and shelf position with the exception of using *Millepora* on offshore reefs.

Based on observations of microhabitat association, all four colour morphs used a variety of different categories based on availability, but did not display any consistent positive selection. Overall, *Acropora*, *Pocillopora*, and dead coral colonies were used consistently among sites, while *Millepora*, *Xeniidae*, soft coral, rubble, sand, pavement, and other sessile organisms were consistently unused (Table 2). Morph 1 consistently used all live coral categories in proportion to availability except for *Millepora* habitats at midshelf reefs. Morph 2 used *Acropora* and *Pocillopora* habitats in equal proportion to availability, however showed mixed usage for other live coral habitats. Morph 3 consistently used *Acropora*, *Pocillopora*, and dead coral colonies in equal proportion to availability while morph 4 used *Acropora* and turf algal habitats and avoided most others (Table 2).

**Table 2:** Microhabitat selectivity indices of the four colour morphs of *P*. *forsteri* across a midshelf to offshore gradient on wave exposed sides of reefs for 14 categories of substrate.

Morphology Shelf position	Zone	n	*Acropora*	*Pocillopora*	*Stylophora*	*Millepora*	*Porites*	Other live hard coral	Xeniidae	Other soft coral	Dead coral colony	Rubble	Sand	Pavement	Turf algae	Other sessileorganisms
**Morph 1**																
Midshelf	Crest	27	= _0.139_	= _0.287_	= _0.172_	U	U	= _0.229_	U	U	= _0.173_	U	U	U	U	U
Midshelf	Slope	36	= _0.236_	= _0.091_	= _0.275_	U	= _0.032_	= _0.069_	U	U	= _0.299_	U	U	U	U	U
Offshore	Crest	34	= _0.116_	-_-0.023_	+_0.24_	= _0.12_	= _0.296_	U	U	U	= _0.152_	U	NA	- _0.00_	= _0.052_	NA
Offshore	Slope	32	= _0.107_	= _0.124_	= _0.234_	= _0.119_	-_-0.013_	= _0.108_	U	= _0.027_	= _0.209_	U	NA	U	= _0.06_	NA
**Morph 2**																
Midshelf	Crest	3	= _0.304_	= _0.696_	U	U	U	U	U	U	U	U	U	U	U	U
Midshelf	Slope	14	= _0.426_	= _0.26_	U	U	= _0.155_	U	U	U	= _0.159_	U	U	U	U	U
Offshore	Slope	15	= _0.203_	= _0.141_	= _0.508_	U	= _0.018_	= _0.014_	U	= _0.092_	= _0.024_	U	NA	U	U	U
**Morph 3**																
Midshelf	Crest	6	= _0.100_	= _0.153_	= _0.171_	U	U	U	U	U	= _0.575_	U	U	U	U	U
Midshelf	Slope	17	= _0.108_	= _0.091_	U	U	U	U	U	= _0.025_	= _0.174_	U	U	U	-_-0.013_	= _0.589_
Offshore	Slope	27	= _0.165_	= _0.159_	= _0.434_	U	U	= _0.038_	U	U	= _0.202_	U	NA	U	-_-0.003_	NA
**Morph 4**																
Midshelf	Slope	6	= _0.322_	= _0.109_	U	U	U	= _0.493_	U	U	U	U	U	U	= _0.076_	U
Offshore	Slope	2	= _0.644_	U	U	U	U	U	U	U	U	U	NA	U	= _0.356_	NA

“=” denotes a category that was used in proportion to its availability,

“+” means the category was used in greater proportion to its availability,

“-“means the category was used at a lower proportion than its availability, and

“U” means the category went unused.

“NA” means the substrate was not present.

Subscripts represent Manly’s standardised selection ratio (B), the probability of one resource unit from the given category being selected if all categories were equally available.

Depth of each zone was: crest = 1 m, slope = 8 m, n = number of individuals analysed.

See Table 1 for substrate abbreviations.

### Activity

**Fig 6:**
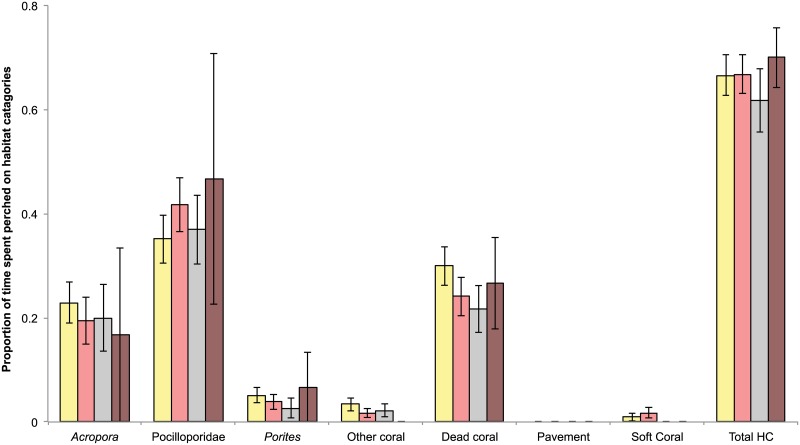
Mean (±SE) proportion of time spent perched on different benthic habitats. Observation period was 10-minutes for four *P*. *forsteri* colour morphs (see Table 3 for number of individuals observed per morph). Yellow = morph 1, red = morph 2, grey = morph 3, brown = morph 4.

*Paracirrhites forsteri* were relatively active, moving from one perching spot to another on average 8 times during a 10 min observation period (Table 3). There was no difference among colour morphs in the number of times an individual moved to another perch or the number of different habitats used during the observation period (approx. 2–3) (Table 3). During this time, all four morphs spent an average of 62% to 70% of their time on live hard corals, predominately on *Acropora* (17% to 23%) and Pocilloporidae (35% to 47%) (Fig 6). In addition, they spent 22% to 30% of their time on dead coral colonies, revealing that ~90% of their time was spent on complex habitats. Individuals often appeared to act threatened by the presence of larger predators (e.g., *Caranx melampygus*) or territorial displays from smaller predators (from mainly *Cephalopholis hemistiktos*) that swam close by. This would result in them taking shelter within or under a habitat rather than perching on top. In 38% of the incidences, morph 1 sheltered in colonies of *Acropora* and 50% in dead coral colonies (Table 4). Morph 2 sheltered mostly in *Acropora* (36%) and Pocilloporidae (43%), while morph 3 sheltered in *Acropora* (50%), dead coral (35%), Pocilloporidae (19%), and *Porities* (5%). Only one incident was recorded for morph 4 and it sheltered in dead coral. *Paracirrhites forsteri* was not observed to shelter in any other category of live hard coral, soft coral, or non-coral substrate (see above for all categories).

**Table 3:** Average (±SE) activity (number of times an individual changed perching position) and the average (±SE) number of habitats each of the four colour morphs used during a 10-minute observation period.

	Activity	Habitats
Morph 1 (30)	7.6 ± SE 0.8	2.7 ± SE 0.1
Morph 2 (31)	9.1 ± SE 0.6	2.5 ± SE 0.1
Morph 3 (21)	8.3 ± SE 0.9	1.9 ± SE 0.2
Morph 4 (3)	8.0 ± SE 0.6	2.3 ± SE 0.3

**Table 4:** Proportional microhabitat use (mean ± SE) for shelter when threatened for each four *P*. *forsteri* colour morph during a 10-minute observation period (all habitats used included).

	Shelter			
	*Acropora*	Pocilloporidae	*Porites*	Dead Coral
Morph 1 (8)	0.38 ± SE 0.18	0.13 ± SE 0.13		0.50 ± SE 0.19
Morph 2 (14)	0.36 ± SE 0.13	0.43 ± SE 0.14	0.07 ± SE 0.07	0.14 ± SE 0.09
Morph 3 (16)	0.50 ± SE 0.13	0.19 ± SE 0.10	0.06 ± SE 0.06	0.25 ± SE 0.11
Morph 4 (1)				1.00 ± SE 0.00

**Fig 7:**
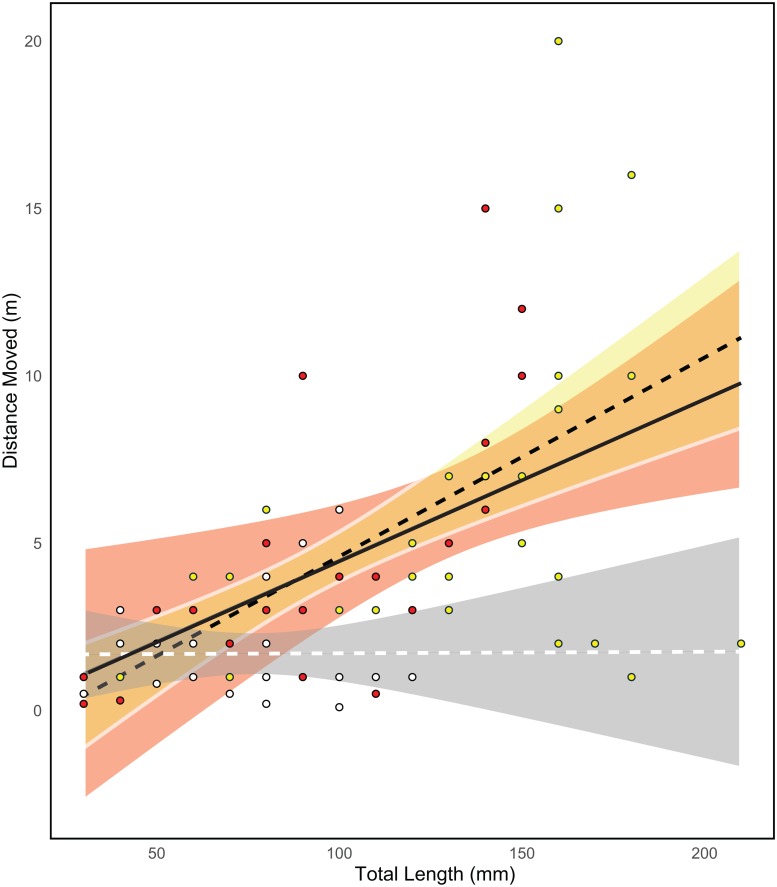
Relationship between total distance moved (m) during 10-minute intervals and body size (total fish length mm). Morph 1 (yellow dots and shading, black dotted line), morph 2 (red dots and shading, black line), and morph 3 (white dots and grey shading, white dotted line). Line represents a best-fit linear line with shaded 95% confidence region.

Individuals of *P*. *forsteri* moved between an average of 0.2 and 20 m during the 10 min observations. Distances covered by individuals during observations positively correlated with body length, with larger bodied individuals moving farther (Fig 7). Individuals of morph 1 and 2 displayed a significant relationship between size (TL) and distance moved within the survey period (P = 0.008, R^2^ = 0.18, and P<0.001, R^2^ = 0.49, respectively). Individuals of morph 3 limited their range to a maximum of 5 m regardless of size, while a relationship could not be drawn for morph 4 due to low numbers of observed individuals (Fig 7). Of the four morphs, morph 1 obtained the largest body size (max. 210 mm) and individuals of all morphs appeared to limit their movement to <5 m when smaller than 130 mm.

## Discussion

Understanding how resource availability and local environmental parameters influence the distribution of organisms is critical in understanding biodiversity and how species will respond to disturbances. All three of the four hawkfish species observed to co-exist in the Red Sea were observed in greater abundance on the exposed side of midshelf and offshore reefs. In addition, abundance and distribution patterns within these zones were not uniform across depths. *Cirrhitichthys oxycephalus* was more commonly encountered at 8 m, while *Paracirrhites forsteri* was present across all depths but more common in deeper zones. *Cirrhitichthys oxycephalus* and *P*. *forsteri* were recorded in greatest densities on the exposed side of midshelf and offshore reefs where they both used live and dead complex habitats in equal proportions to its availability, suggesting that they use a range of habitats but depend on complex microhabitats. Generally, reefs farther from shore exhibit clearer water, higher levels of coral cover and complex habitats [9,48,49].

In this study region, a cross-shelf gradient was observed, whereby inshore reefs and sheltered reef zones contained less overall coral cover and complex coral groups like *Acropora* and Pocilloporidae. Indeed, this study revealed that *P*. *forsteri* are generally not present in areas with less than 18% total live coral cover. *Cirrhitus spilotoceps* (formerly *C*. *pinnulatus* in the Red Sea) was observed on shallow exposed reef crests (1 m depth) where they were mainly associated with non-coral habitats suggesting that they prefer areas of high wave energy irrespective of available habitat. This species appears well adapted for this environment. Individuals are stout, of light and dark brown mottling, and have been reported to wedge themselves within the reef matrix to combat high wave action (see [50]). *Oxycirrhites typus*, known to inhabit gorgonians and black corals in deeper waters [51,52,53], was not recorded, possibly due to the restricted depth of the surveys and scarcity of preferred habitats.

*Paracirrhites forsteri* was the most abundant of the three species and was observed in four distinct colour morphs. This study showed that all morphs occurred sympatrically at a reef scale with overlapping habitats. There were no differences in microhabitat use with all morphs predominantly perched on and sheltered within live and dead coral microhabitats, supporting previous studies’ findings that complex structures are important for these abundant reef mesopredators (see [15,44,45,54,55]). In fact, individuals of different colour morphs were often observed perched together on a single coral head. These microhabitats provide hawkfishes with shelter from predators, a launching platform for hunting, and an observation point for maintaining territories.

There were differences in hawkfish abundance patterns associated with depth. Abundance of morph 2 increased with depth (1–17 m) and morph 3 was more common at a mid depth of 8 m. DeMartini and Donaldson [54] observed a similar trend in the congeneric *P*. *arcatus*, where the white-striped morph increased with depth (1–27 m). However, as with both studies, the mechanisms driving these patterns are unclear. In this study, habitat associations were consistent among morphs, and habitat availability of utilised microhabitats varied little in these zones except for Pocilloporidae, which decreased with depth. It is also possible that depth may influence their ability to avoid visual predators through camouflage and counter shading as a result of a reduction in light levels and certain wavelengths. Difference in pigmentation and countershading may give them an advantage at depth, allowing respective colour morphs to dominate deeper reef regions either as a result of lower predation pressure, increased hunting efficiency, or inter/intraspecific competition [56]. Furthermore, some reef fishes have been shown to actively adjust their hue for ecological advantages (e.g., camouflage, aggressive mimicry), but there is no evidence to date that hawkfish can modify their colour morphs.

Hawkfish are named for their sentinel behaviour of perching atop of reef structures. While this might suggest that these fishes are relatively inactive, individuals of *P*. *forsteri* changed their perching position almost every minute. Hawkfish are known to maintain their movements to feed but also to protect and monitor territories. Within harems, males move about to check on females while females have been noted to protect optimal feeding areas [57]. When *P*. *forsteri* was threatened, either by potential predators or territorial fishes, slight differences among colour morphs were observed in the habitat used for shelter. On all occasions, fish retreated to only *Acropora*, Pocilloporidae, *Porities*, and dead coral colonies for shelter. This was less than the number of categories used for perching and suggests that within a territory, some microhabitats are used for different purposes. Reef structures may provide an ideal vantage point for hunting and defending a territory, but complex coral structures are needed within close range for refuge. Colour morphs 1 and 4 were often observed to retreat to dead coral habitats. Both of these morphs are dark in colouration, and their hue may afford additional camouflage. Similarly, the brown morph of *Pseudochromis fuscus*, a small reef predator, associates in greater proportions with degraded reef habitats than the yellow morph [58]. Our results from longer observation periods support the conclusion that *P*. *forsteri* utilise complex habitats regardless of condition (live and structurally intact dead coral colonies) and these complex hard structures provide physical protection from threats, yet alternative structures may offer the best vantage points for perching [44,45].

Hawkfishes exist on coral reefs within spatial territories that comprise a polygamous social structure [57,59,60]. This study showed that the maximum distance traveled during the observation period for *P*. *forsteri* ranged from 3 m for morphs 2 and 3 to 20 m for morph 1. Size explained about half of the relationship between distance for morphs 2 and 4, with larger individuals moving greater distances than smaller ones. Overall, morph 3 moved relatively small distances compared to the other three morphs, possibly because individuals of this morph were less than 12 cm (TL). Larger individuals of this morph were not observed across the surveys suggesting that there may be demographic differences among morphs or that this morph transitions to another morph above 12 cm. In general, larger hawkfish individuals are potential males with smaller individuals being subordinate females (see [54,59,60]); unfortunately it is not possible to visually identify sex *in situ* and therefore test the effect of sex on behaviour or morph. It is expected that larger males would move greater distances as they have been reported to maintain and defend territories in order to protect mates and prey resources [57]. Therefore, territory size and movement may be dependent on the availability and spacing of complex habitats and play an important role in determining hawkfish assemblages [15,61,62].

Colour polymorphism within the same species is not uncommon in reef fishes (e.g., [37,43,63]), however, the mechanisms that drive colour variants are less clear. For some fish species, the existence of morphs has been explained by geographic range, habitat, aggressive mimicry, sex, ontogeny, or variants of a single polychromatic species [54,58,64,65]. In this study, there was no relationship between body size and colour for *P*. *forsteri*, suggesting that individuals are not changing through ontogenetic stages, however further investigation is needed to validate this. In addition to expanding our understanding of habitat requirements, this study has revealed differences in patterns of distribution and abundance among the four colour morphs. This generates questions of how ecologically different these morphs are. Additional information is needed on demographics, diet, and genetics to gain a better understanding of this complex species and if these morphs are unique to the Red Sea. More information is needed to understand if different morphotypes mix socially and reproductively. For example, can a harem contain individuals of different colour morphs? Is there increased competition among individuals of different colour morphs? Interspecific competition and predation may have synergistic effects on the distributions and abundances of hawkfish. Applying genetic tools could tease apart the more subtle differences among and between the morphs as currently all morphs are considered one species. It may also be possible that individuals can change colour as a result of fitness benefits (e.g., [58,65]).

This study demonstrates that abundance and distribution patterns are shaped by cross-shelf and depth gradients, and wave exposure for hawkfishes, and highlights the unusual occurrence of four distinct colour morphs within a single reef fish species. Interestingly, abundance patterns exist among colour morphs within a species, suggesting ecological differences associated with these four enigmatic morphs. Further investigation is needed to understand these differences, mechanisms, and evolutionary processes involved. Understanding this is fundamental to understanding the ecology of coral reefs and how modifications to environmental parameters will impact associated assemblages.
